# Supplementary material for: CRISPR-Cas9 generated Pompe knock-in murine model exhibits early-onset hypertrophic cardiomyopathy and skeletal muscle weakness
Source: Sci Rep. 2020 Jun 25;10:10321. doi: 10.1038/s41598-020-65259-8 (PMC7316971; doi:10.1038/s41598-020-65259-8)
Supplement: Supplementary file 1 — Supplementary information. [file 41598_2020_65259_MOESM1_ESM.docx]

**Supplementary Figures for:**

**CRISPR-Cas9 generated Pompe knock-in murine model exhibits early-onset hypertrophic cardiomyopathy and skeletal muscle weakness**

Jeffrey Y. Huang^1*^, Shih-Hsin Kan^1^, Emilie K. Sandfeld^1^, Nancy D. Dalton^2^, Anthony D. Rangel^1^, Yunghang Chan^2^, Jeremy Davis-Turak^3^, Jon Neumann^4^, Raymond Y. Wang^5,6

1^CHOC Children’s Research Institute, Orange, CA 92868
^2^Department of Medicine, University of California San Diego, La Jolla, CA 92093
^3^OnRamp BioInformatics, Inc. San Diego, CA 92121
^4^Transgenic Mouse Facility, University of California Irvine, Irvine, CA 92697

^5^Department of Pediatrics, University of California-Irvine School of Medicine, Irvine CA 92697

^6^Division of Metabolic Disorders, CHOC Children’s Specialists, Orange, CA 92868

^*^Corresponding Author: [jhuang@choc.org](mailto:jhuang@choc.org)

**Supplementary Figure 1:**


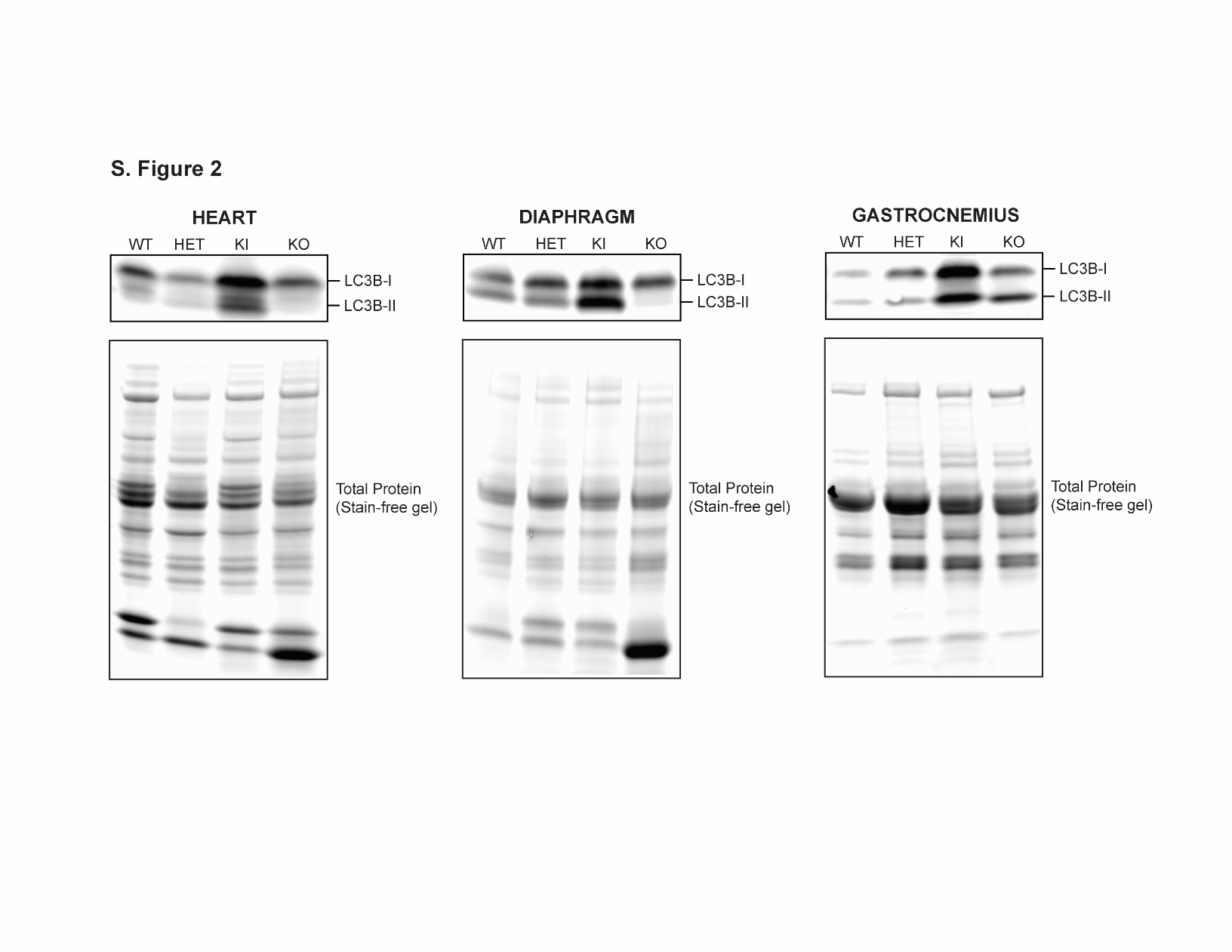


***Gaa*^c.1826dupA^ knock-in mice display increased LC3B-I and LC3B-II protein expression**

Representative anti-LC3B Western blot (top) and stain-free gel (bottom) images of *Gaa*^wt^ (WT), *Gaa*^wt/c.1826dupA^ (HET), *Gaa*^c.1826dupA^ (KI) and *Gaa*^tm1Rabn^ (KO) heart, diaphragm and gastrocnemius tissue protein lysates. LC3B-I and LC3B-II protein levels were measured by densitometric analysis of Western blots probed against a primary LC3B antibody (Sigma, L7543) and normalized to the amount of total protein as determined by densitometric analysis of stain-free gels.

**Supplementary Figure 2:**


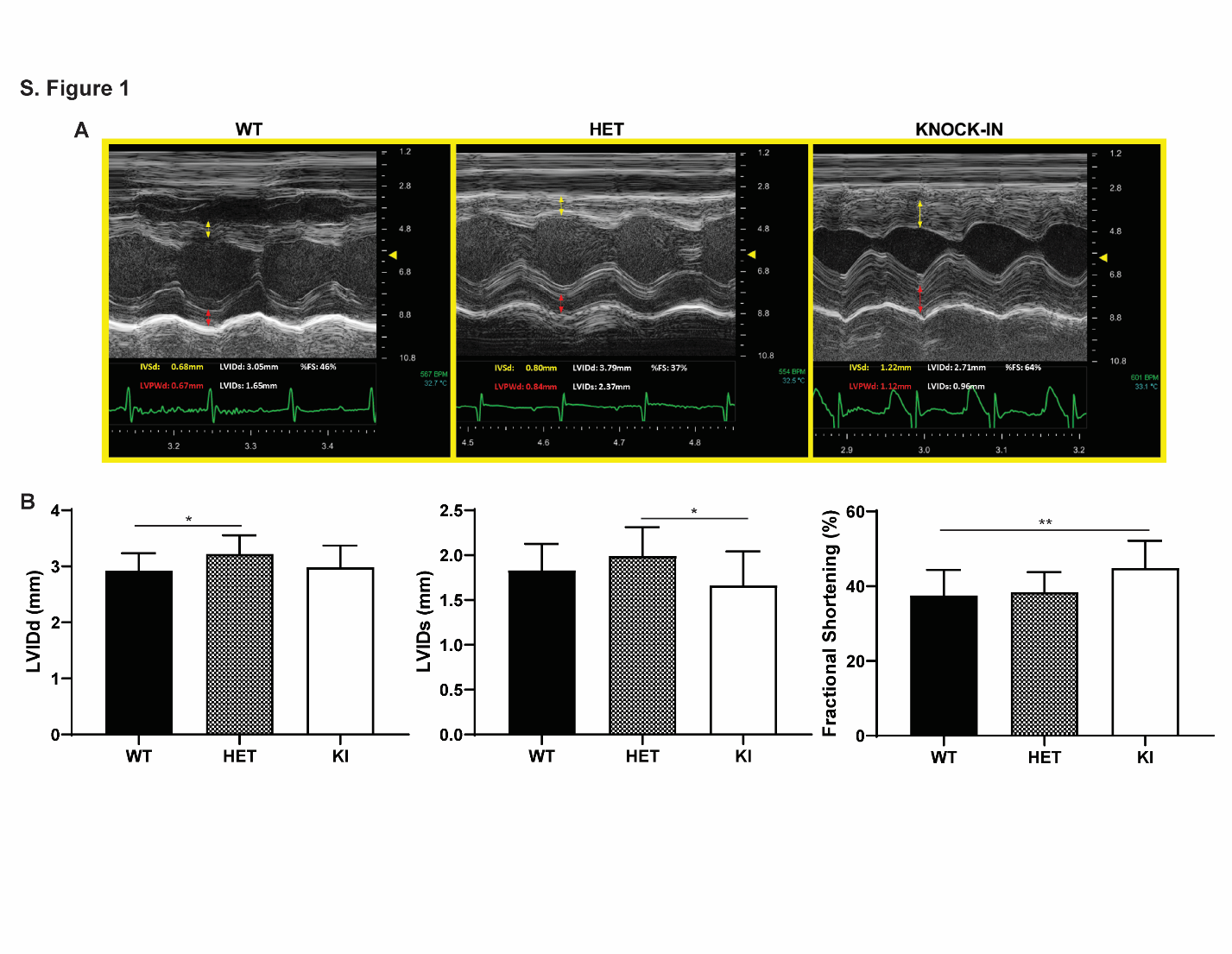


***Gaa*^c.1826dupA^ knock-in mice display altered left ventricular cardiac function**

**(A)** Representative echocardiography images of 3-month old *Gaa*^wt^ (WT), *Gaa*^wt/c.1826dupA^ (HET) and *Gaa*^c.1826dupA^ (KI) mice. Additional anatomical measurements include left ventricular internal diameter end diastole (LVIDd), left ventricular internal diameter end systole (LVIDs) and fractional shortening.

**(B)** LVIDd (left panel), LVIDs (middle panel) and fractional shortening (right panel) measurements in 3- month old *Gaa*^wt^ (WT, n=27), *Gaa*^wt/c.1826dupA^ (HET, n=12) and *Gaa*^c.1826dupA^ (KI, n=13) mice. KI mice exhibit significant alterations in LVIDs (relative to HET) and fractional shortening (relative to WT). All comparisons were analyzed using one-way ANOVA with Tukey post-hoc test. *p<0.05, **p<0.01.
